# Intrauterine Contraceptive Device Migration Presenting as Abdominal Wall Swelling: A Case Report

**DOI:** 10.1155/2011/305914

**Published:** 2011-10-19

**Authors:** Imtiaz Wani, Adil Syed, Muddasir Maqbool, Iftikhar Bakshi, Hilal Bhat, Faheem Ul Hassan Andrabi, Naveed Mohsin

**Affiliations:** ^1^Department of General Surgery, SKIMS, Srinagar, Kashmir, India; ^2^Department of Surgery, SMHS Hospital, Srinagar, Kashmir, India; ^3^Department of General Medicine, SKIMS, Srinagar, Kashmir, India

## Abstract

A number of complications are reported with the use of intrauterine contraceptive devices. These may pursue asymptomatic course or present as an acute abdomen after migration into peritoneal cavity. The authors here are reporting an abdominal wall swelling caused by transuterine migration of a copper intrauterine contraceptive device in a 28-year-old female. An open approach was used, and impacted foreign body was retrieved.

## 1. Introduction

Intrauterine contraceptive devices (IUCD) are regarded as a safe, effective, and economic form of contraception. Possible serious complication associated with its use restricts utilization by a large part of general population [1]. These may migrate inside peritoneal cavity, and pathway for migration is via uterus or fallopian tube. Depending on site and severity of involvement, migration of IUCD present with varying abdominal symptoms and signs or may remain asymptomatic [2]. Radiological investigations can detect asymptomatic migrated IUCD. Even if asymptomatic migrated IUCDs are to be retrieved to prevent serious complications. Retrieval of migrated IUCD may involve open or laparoscopic approach depending on expertise, facilities, and nature of migration. 

## 2. Case History

A 28-year-old female presented with progressive swelling of left paraumbilical region of four-month duration. There was a mild aching pain for last two years for which she used to take medications and get relief. Patient had second full-term normal delivery seven months back, six years after her first delivery.

**Figure 1:**
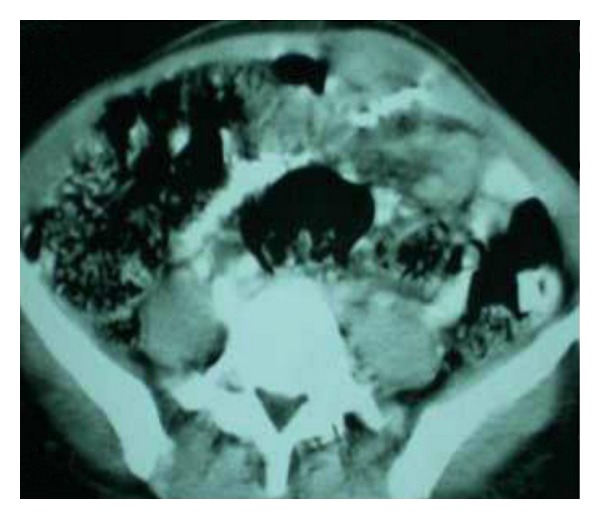
CT scan abdomen showing impacted IUCD.

On retrospective questioning, the patient gave history of having used copper-T as a contraceptive device three years after her first delivery and conceived last delivery with intrauterine device in situ, because of refusal for medical termination of pregnancy in view of religious inhibitions. Neither per speculum examination nor serial pelvic sonography could detect intrauterine contraceptive device during her second pregnancy and was presumed to be expelled without her knowledge as per physician. General physical and systemic examination was normal. Local examination revealed a nontender, firm, mobile 7 × 3.2 × 1.6 centimeter swelling fixed to underlying muscle with free overlying skin. Ultrasonography of abdomen showed marked anterior abdominal wall thickening with IUCD-like structure in it. Chronic inflammatory cells were present on fine needle aspiration of swelling. Computed tomography scan of abdomen showed thickening of anterior abdominal wall with thickened underlying abdominal viscera, and a hyperdense structure impacted in underlying abdominal structures encroaching abdominal wall suggestive of IUCD-like structure was seen (Figure 1).

**Figure 2:**
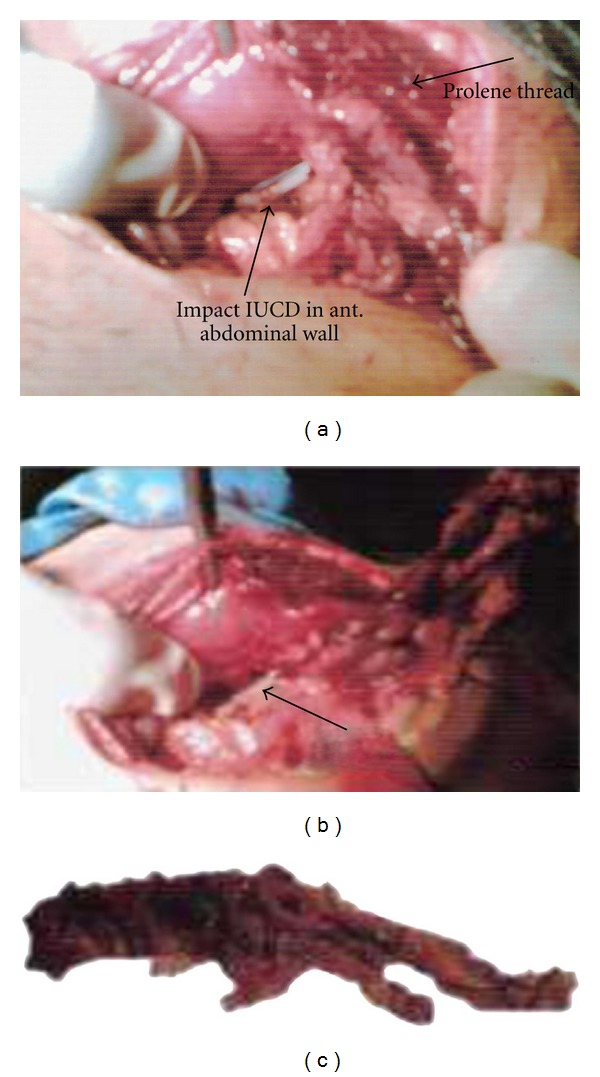
(a) and (b) Intraoperative figure showing impacted IUCD. (c) Showing retrieved IUCD.

The presence of ectopic IUCD was likely to have generated chronic inflammation only in its immediate surroundings, and tight intraabdominal adhesions preventing the laparoscopic approach. Laparotomy was done, and IUCD logged in rectus muscle wrapped with omentum from site of perforating uterine wall was seen (Figures 2(a), 2(b), and 2(c)). The device was removed along with wrapped omentum with the repair of tissues done. Postoperative period was uneventful. Patient is regularly attending our follow-up clinics.

## 3. Discussion

In developing countries, intrauterine contraceptive device forms one of the integral parts of family planning methods. These are considered as one of the cost-effective contraceptive devices. A range of intrauterine contraceptive devices are offered for measures of contraception. Various copper contraceptive commonly in use are copper T 200, copper T, multiload copper—250, and multiload copper—375. The design, copper content, method of placement, and timing of insertion determine profile of side effects. Risk factors for migration are use in nullipara, postpartum or postabortion insertion, faulty technique of insertion, and irregular followup [3]. Migration is associated with a significantly higher rate in immediate postpartum insertion of intrauterine device. Migration can be incomplete or complete. In former type, the device remains attached to the myometrium whereas, in complete migration, the device may be situated in any site in abdomen. Pelvic complications reported with the use of intrauterine contraceptive device are in the form of dysmenorrhea, pelvic inflammatory disease, septic abortion, and hydrosalpinx. Perforation of the uterine wall and transuterine migration of intrauterine contraceptive device into abdominal cavity are rare and can lead to dreadful complications [4]. Perforation of uterus occurs in 1/350 to 1/2500 insertions [5]. Inert positioning, fragility of uterine wall due to recent birth, abortion, and pregnancy are contributory to the possibility of uterine perforation [6]. After perforating uterine wall intrauterine contraceptive device can have migration to colon, wall of iliac vein, bladder, appendix, omentum, perirectal fat, retroperitoneal space, pouch of douglas, and ovaries [7–10]. Rarely, IUCD migrated can be located in lower anterior abdominal wall [11]. In bladder, they lead to calculi formation [12]. Regular self-examination, investigation of persistent pain, or disappearance of strings may detect migration early [13]. X-ray abdomen, ultrasonography, and computed tomography scan are usually used for diagnosis. Plain X-ray is useful and can detect migration of intrauterine contraceptive device. Ultrasonography and computed tomography scan are adjuncts in locating site of impaction. Transvaginal ultrasonography visualizes the IUD located outside the uterus. There are proponents of leaving migrated asymptomatic intrauterine contraceptive device as such, but not well supported in literature. All the copper-containing devices require laparotomy for removal because of an omental or peritoneal reaction incited with their presence [14]. Detection of asymptomatic migrated intrauterine contraceptive device necessitates retrieval in order to discourage psychosomatic symptomatology, commonly associated with forgotten devices and prevention of future grave complications [15]. Laparotomy, colpotomy, and laparoscopy are treatment options available for migrated foreign bodies. Laparoscopy has advantage that it enables localization of the intrauterine contraceptive device and full lesion assessment [16]. Parietoepiploic adhesions and IUCD impacted in gut wall limit generous use of laparoscopy in salvage.

## 4. Conclusion

A regular followup for detection of misplacing of intrauterine device is stressed as it can have unusual presentation. Migration to anterior abdominal wall presenting as swelling could be considered as differential diagnosis of abdominal swelling.
